# Inhibition of HDAC6 activity in kidney diseases: a new perspective

**DOI:** 10.1186/s10020-018-0027-4

**Published:** 2018-06-26

**Authors:** Ben Ke, Yanxia Chen, Wei Tu, Ting Ye, Xiangdong Fang, Liping Yang

**Affiliations:** 1 Department of Nephrology, The Second Affiliated Hospital of Nanchang University, Nanchang, 330006 Jiangxi China; 2 Department of Endocrinology, The Affiliated Tongji Hospital of Huazhong University of Science and Technology, Wuhan, 430000 Hubei China; 3 Department of Intensive Care Unit, The First Affiliated Hospital of Gannan Medical University, Ganzhou, 341000 Jiangxi China; 4 Department of Breast, Jiangxi Cancer Hospital, Nanchang, 330006 Jiangxi China; 5 Nanchang, People’s Republic of China

**Keywords:** Histone deacetylase 6, Autosomal dominant polycystic kidney disease, Renal fibrosis, Lupus nephritis, Acute kidney injury

## Abstract

Histone deacetylase 6 (HDAC6), a cytoplasmic enzyme that plays important roles in many biological processes, is one isoform of a family of HDAC enzymes that catalyse the removal of functional acetyl groups from proteins. HDAC6 stands out from the other members of this family because it almost exclusively deacetylates cytoplasmic proteins and exerts deacetylation-independent effects, which has led to the successful development of relatively isoform-specific inhibitors of its enzymatic action. Numerous studies have recently demonstrated that HDAC6 expression and activity are increased in kidney disease, such as autosomal dominant polycystic kidney disease (ADPKD), renal fibrosis, and acute kidney injury (AKI), among others. Moreover, HDAC6 inhibitors have been investigated for use in treating these diseases. In fact, HDAC6 inhibitors effectively limit the progression of kidney diseases, suggesting that targeting HDAC6 may provide a novel treatment approach. However, the primary challenge in developing HDAC6-targeted therapies is understanding how the renoprotective effect of NDAC6 inhibitors can be selectively harnessed. Here, we discuss the unique function of HDAC6 and recapitulate the alluring potential of its inhibitors in kidney diseases.

## Background

**Fig. 1:**
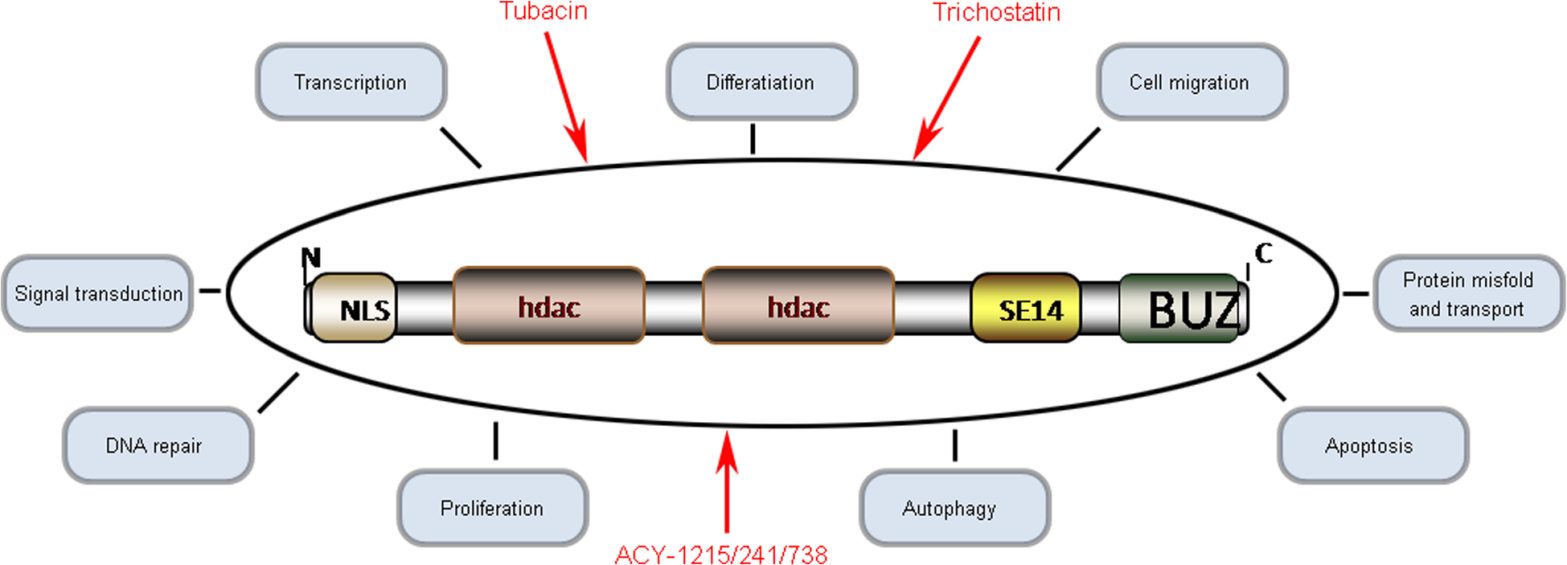
Schematic diagram of the structure and pleiotropic functions of HDAC6 protein

Histone deacetylase 6 (HDAC6) is a cytoplasmic protein that potentially regulates several cellular functions through deacetylase-dependent and/or deacetylase-independent mechanisms (Valenzuela-Fernandez et al. 2008). HDACs are a group of enzymes that play important roles in multiple cellular processes by removing the acetyl group from histone or nonhistone proteins (Li 2011). Based on their homology to yeast orthologous, mammalian HDACs are categorized into 4 groups: class I HDACs (HDACs1, 2, 3, and 8), class II HDACs (HDACs 4, 5, 6, 7, 9, and 10), class III HDACs (SIRT1–7), and class IV HDACs (HDAC11) (Batchu et al. 2016). HDAC6 was first discovered in 1999 because of its homology to HDAC1 (Grozinger et al. 1999; Verdel and Khochbin 1999). The gene encoding HDAC6 is localized to the sub-band border of chromosome Xp11.22–23 in humans (Voelter-Mahlknecht and Mahlknecht 2003). Unlike most HDAC isoforms, HDAC6 contains a Ser Glu-repeat domain (SE14) that acts as a cytoplasmic retention signal and mediates its stable anchorage in the cytoplasm (Bertos et al. 2004). At the N-terminus of the enzyme, there is a nuclear localization signal (NLS) that enables the deacetylase to shuttle between the nucleus and the cytoplasm (Liu et al. 2012a). HDAC6 is unique in that it has duplicated deacetylase domains as well as a C-terminal binder of the ubiquitin zinc (BUZ) finger domain, which binds ubiquitin (Seigneurin-Berny et al. 2001) (Fig. 1). Although this enzyme is named HDAC6, it does not exert detectable deacetylase activity towards histones in vivo (Zhang et al. 2003; Tran et al. 2007), while it deacetylates histones in vitro (Zhang et al. 2006). The most characterized substrates of HDAC6 include α-tubulin, heat shock protein 90 and cortactin (Zhang et al. 2003). As a key modulator, HDAC6 has been implicated in many cellular processes, including proliferation (Valenzuela-Fernandez et al. 2008), autophagy (Lin et al. 2017), apoptosis (Bitler et al. 2017), and DNA repair (Namdar et al. 2010) (Fig. 1). Furthermore, HDAC6 inhibitors have shown powerful potential therapeutic effects in multiple diseases, such as cancer, neurodegenerative diseases, cardiovascular diseases, and kidney disease, among others *(Batchu* et al. 2016*)*.

HDAC6 has recently emerged as a vital cytokine in kidney diseases. Growing evidence has demonstrated that the expression and activity of HDAC6 are increased in various kidney diseases (Shi et al. 2017; Choi et al. 2015; Cebotaru et al. 2016), and inhibiting HDAC6 has mitigated the progression of kidney injury. In this review, we focus on the role of HDAC6 in the pathology of kidney diseases and recapitulate the alluring potential of its inhibitors, which may aid in the development of HDAC6-targeted therapies for kidney diseases.

### HDAC6 and ADPKD

Autosomal dominant polycystic kidney disease (ADPKD) is one of the most common hereditary disorders in humans, affecting 1/500 individuals in the United States (Gabow 1993). The hallmark of ADPKD is the development of multiple bilateral renal cysts that replace normal renal parenchyma, resulting in end-stage renal disease (ESRD) in approximately 50% of individuals with ADPKD by the age of 50 (Chebib et al. 2015). Most cases of ADPKD are caused by mutations in one of two genes: *PKD1*, accounting for 85–95% of cases, and *PKD2*, accounting for most of the remaining cases (Peters and Sandkuijl 1992). Aberrant Ca2+ signalling and cyclic adenosine monophosphate (cAMP) signalling are the leading causes of ADPKD (Chebib et al. 2015), and aberrant Ca2+ metabolism causes a switch to a proliferative cAMP-dependent phenotype in ADPKD (Chang and Ong 2013). The hypothesis is that Ca2+ restriction in ADPKD cells causes cAMP-dependent activation of the B-Raf/mitogen-activated protein kinase (MEK)/extracellular signal-regulated kinase (ERK) pathway, resulting in increased cell growth (Yamaguchi et al. 2004). Similarly, increased Ca2+ influx into ADPKD cells restores normal cAMP signalling, thereby reducing cell growth (Yamaguchi et al. 2006). However, this hypothesis remains debated, and thus, the function of PC1 and PC2 in Ca2+ signalling is controversial (Chebib et al. 2015).

HDAC6 promotes cyst formation and disease by enhancing intracellular cAMP in ADPKD. Abnormal proliferation of the cyst-lined epithelium and increased intra-cystic fluid secretion via the cystic fibrosis transmembrane conductance regulator (CFTR) may contribute to cyst growth in ADPKD (Ramasubbu et al. 1998; Hanaoka et al. 1996). CFTR, a cAMP-activated chloride channel, is expressed in the apical epithelia in ADPKD cystic tissue, and its function can be measured by cAMP-mediated activation of chloride currents (Hanaoka et al. 1996). Cebotaru et al. found that the HDAC6-specific inhibitor tubacin inhibited cystic cell proliferation and reduced cAMP-triggered activation of CFTR in canine renal epithelial cells (Cebotaru et al. 2016). Consistently, Yanda and colleagues also found that treatment with ACY-1215, a specific HDAC6 inhibitor, slowed cyst growth in a mouse model of ADPKD by lowering cAMP levels (Yanda et al. 2017a). Interestingly, Yanda et al. found that HDAC inhibition decreased intracellular resting Ca2+ and increased ATP-simulated Ca2+ release in PC1 knockout (KO) cells. HDAC6 inhibition reduced the release of Ca2+ from the ER induced by thapsigargin, an inhibitor of the endoplasmic reticulum, Ca2 + -ATPase. HDAC6 inhibition and treatment of cells with the intracellular Ca2+ chelator BAPTA-AM reduced cAMP levels in PC1 KO cells (Yanda et al. 2017b). The poorly understood roles of PC1 and PC2 may responsible for this paradoxical phenomenon.

HDAC6 exaggerates ADPKD by upregulating epidermal growth factor receptor (EGFR) activity (Liu et al. 2012b). EGFR, which is also known as ErbB receptor tyrosine kinase, plays important roles in renal development, renal electrolyte homeostasis and tubule repair following injury (Melenhorst et al. 2008). Studies have shown that inhibition of EGFR tyrosine kinase activity, either genetically or pharmacologically, significantly reduces renal cyst formation and improves renal function in rodent models of PKD (Melenhorst et al. 2008; Sweeney et al. 2000; Torres et al. 2003), indicating that persistent EGF signalling is a primary factor of disease progression in PKD. HDAC6, a microtubule-associated α-tubulin deacetylase, exhibits increased expression and activity in Pkd1 mutant mouse embryonic kidney cells (Liu et al. 2012b). Liu et al. (Liu et al. 2012b) found that targeting HDAC6 with a general HDAC inhibitor, trichostatin, or a specific HDAC6 inhibitor, tubacin, increased the acetylation of α-tubulin and downregulated the expression of EGFR in Pkd1 mutant renal epithelial cells. In addition, inhibition of HDAC activity decreased the phosphorylation of ERK1/2, a downstream target of the EGFR axis, and normalized EGFR localization from apical to basolateral in Pkd1 KO mouse kidneys (Liu et al. 2012b). Thus, these authors suggested that Pkd1 mutation-induced upregulation of HDAC6 might slow the trafficking of EGFR from early endosomes to late endosomes along microtubules for degradation through deacetylation of α-tubulin, leading to phosphorylation of ERK1/2 to facilitate cyst formation (Liu et al. 2012b). Additionally, EGF contributes to ADPKD by inducing abnormal activation of the Wnt/β-catenin-dependent pathway and nuclear translocation of β-catenin (Li et al. 2008). It has been shown that HDAC6 regulates EGF-induced nuclear translocation of β-catenin (Deribe et al. 2009). EGF receptor activity is increased, and the receptor is mis-localized to the apical membrane in *Pkd1* KO mice, whereas inhibition of HCAC6 activity in *Pkd1* KO mice restores EGF localization to the basolateral cell membrane (Liu et al. 2012b).

**Fig. 2:**
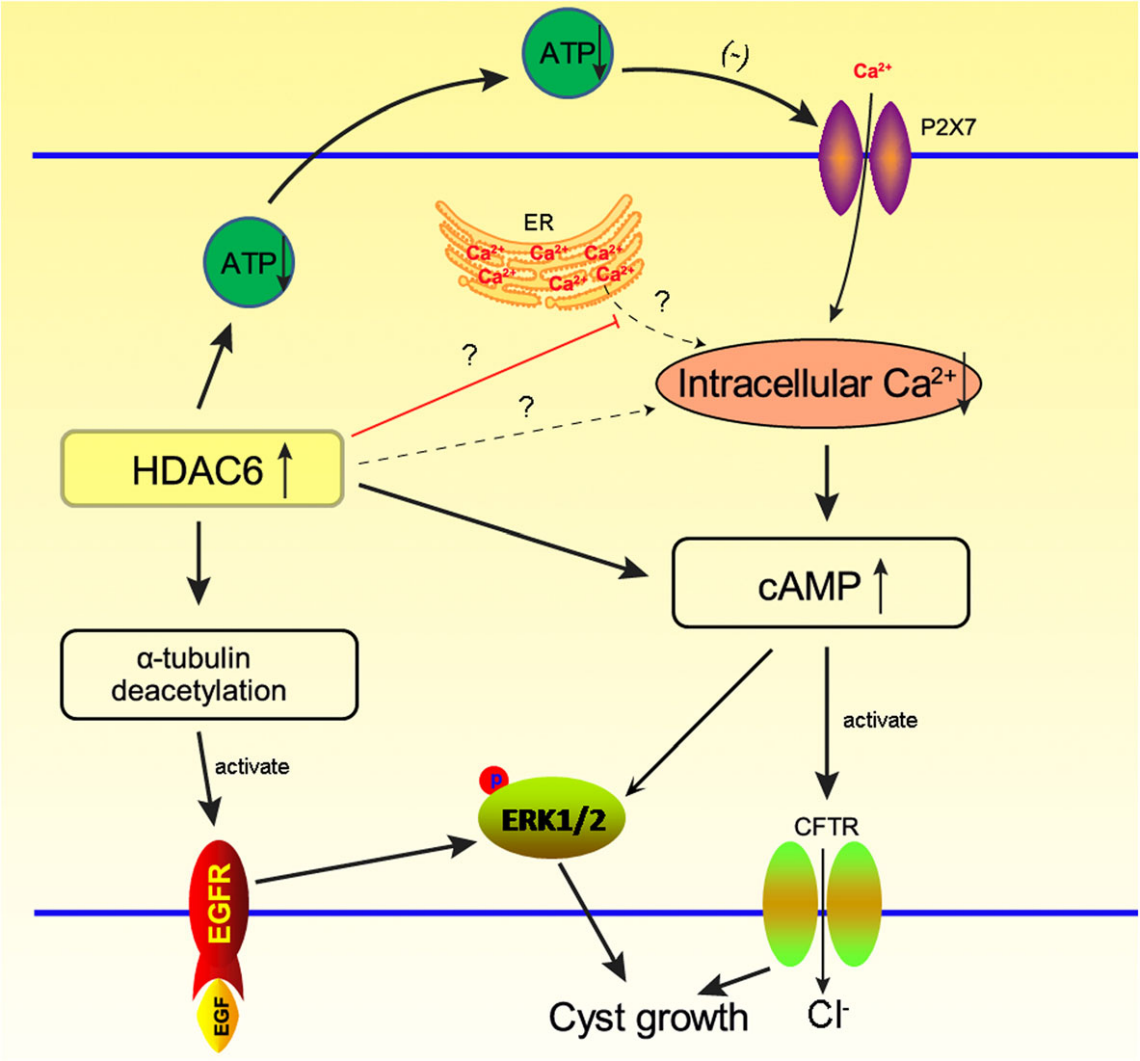
Schematic presentation of the signalling pathways by which HDAC6 contributes to cyst growth by promoting cell proliferation and fluid secretion induced by aberrant Ca2+ signalling, abnormal cAMP signalling, and prolonged EGFR signalling

Collectively, HDAC6 contributes to cyst growth by promoting cell proliferation and fluid secretion induced by aberrant Ca2+ signalling, abnormal cAMP signalling, and prolonged EGFR signalling (Fig. 2).

### HDAC6 and renal fibrosis

Renal fibrosis, characterized by fibroblast proliferation and the accumulation of extracellular matrix (ECM), is the endpoint of chronic kidney disease. Connective tissue growth factor (CTGF), implicated in the formation of ECM, including the ECM proteins fibronectin and collagen, is considered to be a molecular hallmark of renal fibrosis (Gao et al. 2008). Renal fibrosis is a complicated process, and multiple cellular cytokines are involved in the onset of renal fibrosis. As a participant in the process of fibrosis through activation of Smad2 and Smad3 phosphorylation, TGF-β/Smad signalling is considered to be the key regulator in renal fibrosis (Wang et al. 2014).

HDAC6 contributes to renal fibrosis through regulation of epigenetic histone modification and Smad3-dependent fibrotic genes (Choi et al. 2015). A previous study showed that the expression of HDAC6 was increased in a hypertensive kidney damage mouse model (Choi et al. 2015), and inhibition of HDAC6 or small interfering RNA against HDAC6 attenuated hypertensive stimuli-induced renal fibrosis and inflammation (Choi et al. 2015). Choi et al. investigated the fibrotic mechanism of HDAC6 and found that HDAC6 may participate in the regulation of epigenetic histone modification and facilitate phospho-Smad2/3 to Smad3 binding elements in fibrosis-associated gene promoters (Choi et al. 2015). Additionally, Shan et al. revealed a novel function of HDAC6 in epithelial-mesenchymal transition (EMT) by intercepting the TGF-β-SMAD3 signalling cascade (Shan et al. 2008). Aberrant EMT has been well documented in renal fibrosis. Shan and colleagues found that TGF-β1-induced EMT is accompanied by HDAC6-dependent deacetylation of α-tubulin. Importantly, inhibition of HDAC6 attenuated TGF-β1-induced EMT markers, such as aberrant expression of epithelial and mesenchymal peptides, as well as the formation of stress fibres. Reduced expression of HDAC6 also impaired SMAD3 activation in response to TGF-β1. Conversely, inhibition of SMAD3 activation substantially impaired HDAC6-dependent deacetylation of α-tubulin as well as the expression of EMT markers (Shan et al. 2008).

Finally, a recent study reported that unilateral ureteral obstruction disrupted microtubules, accompanied by less reduction of HDAC6 and α-tubulin acetyl transferase, which acetylates tubulin, a component of the microtubule; this finding means that HDAC6 may lead to renal fibrosis by inducing deacetylation of α-tubulin (Noh et al. 2018). Hence, HDAC6 may be a valuable therapeutic target for the treatment of renal fibrosis.

### HDAC6 and lupus nephritis

Systemic lupus erythematosus (SLE) is an autoimmune disease in which the immune system produces autoantibodies against normal healthy tissue or cellular components to form immune complexes that are deposited in various tissues and subsequently induce inflammation, leading to tissue damage (Apostolidis et al. 2011). Lupus nephritis (LN) occurs in approximately 50% of SLE patients and is a major cause of morbidity and mortality due to this disease (Almaani et al. 2017). The pathogenesis of LN is complicated. Studies using lupus-prone mice have demonstrated that ablation of plasmacytoid dendritic cells (pDCs), a major source of interferon-α (IFN-α), prevented LN progression, reduced autoreactive T and B cell activation, and decreased autoantibodies in circulation and renal deposition (Sisirak et al. 2014; Rowland et al. 2014). Several studies have also shown a critical role of IFN-α in LN (Abdel Galil et al. 2017; Ji et al. 2015). Nevertheless, the role of IFN-α in LN is still unknown.

HDAC6 may play an active role in LN by increasing IFN-α levels. Several studies have recently provided evidence that epigenetic factors, including acetylation of histones and non-histone proteins, play crucial roles in the initiation and development of SLE, including LN (Hedrich 2017). Ren et al. found that HDAC6 expression is increased in animal models of SLE and that inhibition of HDAC6 decreased the disease. Furthermore, these authors examined the function of the HDAC6 inhibitor ACY-738 in LN and revealed that the IFN-α-producing ability of pDCs was decreased along with immunoglobulin isotype switching and the generation of pathogenic autoantibodies. Additionally, renal tissue showed decreased immunoglobulin deposition and reduced inflammation, as determined by glomerular and interstitial inflammation (Ren et al. 2017). This study indicates that HDAC6 is indeed involved in the development of LN, although further studies are needed to clarify the function of HDAC6 in the immune response.

Notably, Ren et al. concluded that HDAC6 inhibition may suppress B cell development and responses (Ren et al. 2017), which plays an essential role in the pathogenesis of LN (Suso et al. 2018). Similarly, primary membranous nephropathy (MN) is one of the most common types of autoimmune nephritis, in which phospholipase A2 receptor (PLA2R) and thrombospondin type-1 domain-containing 7A (THSD7A) are the two major autoantigens (Beck Jr et al. 2009; Tomas et al. 2014). Autoreactive B-cell clones can produce anti-PLA2R and anti-THSD7A antibodies, which interact with their corresponding antigens, resulting in the deposition of immune complexes in primary MN (Ruggenenti et al. 2017). Taken together, HDAC6 inhibitors may exert a renoprotective role in both LN and primary MN; however, no study has reported this effect in primary MN.

### HDAC6 and AKI

Acute kidney injury (AKI), which is characterized by a rapid decline in the glomerular filtration rate, is a serious clinical problem. AKI is not only associated with high rates of mortality but also an increased risk of chronic kidney disease (Geng et al. 2015). Rhabdomyolysis, one of the major causes of community-acquired AKI, accounting for 5–15% of cases, is induced by different conditions, including crush injuries, severe trauma, intense physical exercise and some medications and illicit drugs, such as cocaine (Komada et al. 2015; Panizo et al. 2015). Rhabdomyolysis induces multiple deleterious effects on the kidney, including apoptosis, inflammation, oxidative stress, vasoconstriction, and tubular obstruction (Panizo et al. 2015; Humphreys et al. 2008). During these pathological processes, multiple signalling pathways and numerous genes involved in cell death and inflammatory responses are activated and/or upregulated (Geng et al. 2015). However, the detailed mechanism responsible for the pathogenesis of rhabdomyolysis-induced AKI is poorly understood. Understanding the molecular basis of these processes will aid in devising therapeutic strategies to treat AKI.

Inhibition of HDAC6 protects against ischaemic stroke and prolongs survival after sepsis in animal models (Wang et al. 2016; Li et al. 2015). Consistently, increased HDAC6 expression was observed in the cytoplasm of renal tubular cells, which commonly results in rhabdomyolysis-induced AKI, in a sepsis animal model (Shi et al. 2017). Administration of tubastatin A, significantly reduced the serum creatinine and blood urea nitrogen levels and attenuated renal tubular damage in the injured kidneys (Shi et al. 2017). Moreover, HDAC6 inhibition also resulted in decreased expression of NGAL, an injury marker of renal tubules, reduced apoptotic cells and decreased the expression of proinflammatory cytokines in the kidney after acute injury (Shi et al. 2017; Tang et al. 2018). Furthermore, HDAC6 inhibition reduced the level of oxidative stress by suppressing malondialdehyde (MDA) and preserved the expression of superoxide dismutase (SOD) in the injured kidneys (Shi et al. 2017). Recently, Yuying Feng et al. found that HDAC6 inhibitor shows renal protection via the reduction of endoplasmic reticulum (ER) stress-mediated apoptosis in tubular epithelial cells of rhabdomyolysis-induced AKI (Feng et al. 2018). Collectively, HDAC6 may accelerate AKI, mainly in rhabdomyolysis-induced AKI, by inducing oxidative stress, inflammation and ER stress.

## Conclusion

HDAC6 plays a aggravating role in kidney diseases, and up-regulation of HDAC6 could exacerbate kidney diseases. In ADPKD, a role of HDAC6 in cytogenesis has been identified, as HDAC6 facilitates the progression of ADPKD by regulating Ca2+ signalling, cAMP signalling and EGFR endocytic trafficking and degradation. Moreover, treatment of ADPKD with HDAC6 inhibitors is effective, suggesting that these molecules are promising drug candidates for alleviating ADPKD. Furthermore, HDAC6 is closely related to other kidney diseases, including LN and AKI. Given its function in immune responses, HADC6 may participate in the onset of primary MN; however, additional studies are needed to confirm this idea.
